# A First-Principles Study of Mechanical and Electronic Properties of Cr_0.5-x_Al_0.5_TM_x_N Hard Coatings (TM = Ti, V, Y, Zr, Hf, and Ta)

**DOI:** 10.3390/ma17051070

**Published:** 2024-02-26

**Authors:** Weike Dai, You Zou, Jiong Wang, Yue Su, Donglan Zhang

**Affiliations:** 1 State Key Laboratory of Powder Metallurgy, Central South University, Changsha 410083, China; aliang1421@163.com (W.D.); 213311014@csu.edu.cn (Y.S.); 203301046@csu.edu.cn (D.Z.); 2 Information and Network Center, Central South University, Changsha 410083, China; zouyou@csu.edu.cn

**Keywords:** doped CrAlN, first-principles calculations, mechanical properties, electronic properties

## Abstract

The structural, mechanical, and electronic properties of cubic Cr_0.5-x_Al_0.5_TM_x_N, doped with TM (transition metal) elements (TM = Ti, V, Y, Zr, Hf, and Ta) at low concentrations (x = 0.03 and 0.06), was investigated by first-principles calculations. The results of the structural properties calculations reveal that the addition of Ti, Y, Hf, Zr, and Ta expand the volume, while V has the opposite effect. All doped compounds are thermodynamically stable, and Cr_0.5-x_Al_0.5_TM_x_N with TM = Ti is energetically more favorable than other doped compounds. At the same doping concentration, Cr_0.5-x_Al_0.5_V_x_N possesses the highest stiffness, hardness, and resistance to external forces due to its greatest mechanical properties, and Cr_0.5-x_Al_0.5_Ta_x_N possesses the highest elastic anisotropy and the lowest Young’s modulus. Substituting Cr atoms with TM atoms in a stepwise manner results in a decrease in the bulk modulus, shear modulus, Young’s modulus, and theoretical hardness of Cr_0.5-x_Al_0.5_TMxN, while increasing its toughness. Based on the calculation results of the total and partial density of states of Cr_0.5_Al_0.5_N and Cr_0.47_Al_0.5_TM_0.03_N, all compounds exhibit metallic behavior as indicated by the finite density of states at the Fermi level. The contribution of Ti-3d, V-3d, and Ta-3d orbitals at Fermi level is significantly higher than that of other TM atoms, resulting in a more pronounced metallic character for Cr_0.47_Al_0.5_Ti_0.03_N, Cr_0.47_Al_0.5_V_0.03_N, and Cr_0.47_Al_0.5_Ta_0.03_N.

## 1. Introduction

Transition metal nitride coatings are widely used in machining tools, material forming molds, diffusion barriers, and other applications due to their good mechanical properties, exceptional oxidation resistance, and thermal stability. Among them, physical vapor deposited (PVD) CrAlN is one of the most promising wear-resistant protective coatings. However, CrAlN has difficulty in maintaining its excellent properties at high temperatures. Studies have shown that temperatures exceeding 1000 °C lead to the release of N_2_ and the gradual decomposition of CrAlN into cubic (bcc-) Cr, wurtzite (w-) AlN, and a small amount of hexagonal Cr_2_N [1,2,3]. Simultaneously, CrAlN coatings exhibit a high friction coefficient, leading to wear and heat concentration issues that can impact their wear resistance [4,5]. It makes CrAlN coatings limited in industrial applications. Recent studies have demonstrated that the incorporation of various elements such as Nb [6], Mo [7], Y [8,9,10,11,12,13,14,15,16,17,18,19,20,21], Ni [22], Ta [23,24], Hf [25], V [5,21,26,27,28,29], Zr [13,15,30,31], Ti [21,32,33,34,35], Si [36], and B [37] can enhance the comprehensive properties of coatings. For example, alloying element with Y has been reported to enhance the thermal stability, mechanical properties, tribological performance, oxidation resistance, and corrosion resistance [6,9,13,19,20]. However, it was demonstrated that mechanical properties, wear resistance, and oxidation resistance decreased with excess Y addition [9,10,12,14]. The incorporation of Ta into CrAlN coatings has been found to enhance thermal stability [23]. Nevertheless, it was observed that the addition of Ta had a detrimental impact on the oxidation resistance of CrAlN coatings [23]. According to Tillmann [25], a decrease in hardness was observed with an increase in Hf content, due to the structural changes. A small amount of Hf doping could increase the oxidation resistance of CrAlN [25]. Delgado [27] evaluated the friction and wear properties of V-rich AlCrVN, and found that V addition increased the hardness and retained the oxidation resistance for annealing treatments up to 460 °C. Alloying with Zr into CrAlN strengthened the hardness, thermal stability, and tribological performance. However, the addition of Zr has been found to have an adverse impact on oxidation resistance of CrAlN [13,20]. CrAlN coatings alloyed with Ti, with a tuned composition, demonstrated enhanced thermal stability, hardness, wear resistance, and oxidation resistance compared to unalloyed CrAlN coatings [32,33,34,35].

Moreover, first-principles calculations based on the density function theory (DFT) have the capability to design new materials, providing valuable guidance for experimental endeavors [38,39]. In a study by Ou [40], the effects of transition metals (Y, Zr, Nb, Hf, Ta) on the elastic and structural properties of TiN were investigated. The calculation results showed favorable agreement with both theoretical predictions and experimental observations. Rovere [16] conducted experiments and computations to explore the influence of Y on the phase stability and mechanical properties of CrAlN coatings. The author found that the substitution of Cr by Y enhanced the phase stability owing to the depletion of anti-bonding states, while substituting Al with Y decreased the phase stability owing to the lattice strain. The study of Hollerweger [24] focused on growth morphology, mechanical properties, and structure. The calculation result showed that with an increase in Ta, Young’s moduli significantly decreased from around 500 GPa to 375 GPa, which is in good agreement with the experimental result.

Our previous study has used first-principles calculations based on atomic scale to predict the structure, surface, and electronic properties of cubic (c-) CrAlN in the antiferromagnetic (AFM) state, with the stable phase below the Néel temperature. It has been observed that the (001) surface terminated with Cr-Al-N is energetically favorable and O is adsorption favored for Cr instead of Al owing to the stronger Cr-O bond strength than that of Al-O [41]. However, to date, there have been few studies investigating the effects of transition metal elements on the structural and mechanical properties of CrAlN coatings using first-principles calculations. In the present work, first-principles calculations based on the density functional theory (DFT) are employed to investigate the structural, mechanical, and electronic properties of c-Cr_0.5_Al_0.5_N at low transition metal doping concentration in the AFM state. The research is more concerned about mechanical properties, which were computed based on the high-efficiency stress–strain method. The total and partial density of states (TDOS and PDOS, respectively) are employed to investigate the electronic properties.

## 2. Methodology

### 2.1. First-Principles Calculations

**Figure 1:**
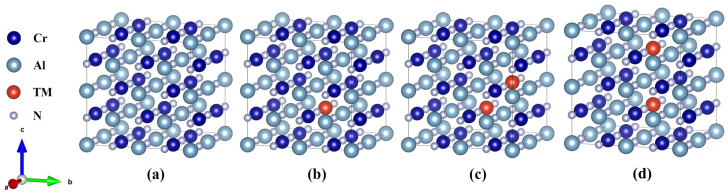
The supercells of (**a**) Cr_0.5_Al_0.5_N, (**b**) Cr_0.47_Al_0.5_TM_0.03_N, and (**c**) Cr_0.44_Al_0.5_TM_0.06_N (neighbor) and (**d**) Cr_0.44_Al_0.5_TM_0.06_N (second-nearest neighbor), where TM = Ti, V, Y, Zr, Hf, and Ta.

The crystal structure of CrAlN in the AFM state investigated in our previous work belongs to NaCl-type face centered cubic (FCC) with space group $\mathrm{Fm}\bar{3\;m}$ (No. 255) [41]. In this work, all calculations are carried out with 2 × 2 × 2 supercells, which contain 32 N atoms and 32 metal atoms. The models are demonstrated in Figure 1a,c. The Cr_0.5_Al_0.5_N (shown in Figure 1a) supercell is set to the original supercell. After that, both Al and Cr atoms are likely to be replaced by the transition metal atom including Ti, V, Y, Zr, Hf, and Ta, which will be referred to as TM atom in the following sections. To consider the atomic arrangement around the replaced atoms, it is only surrounded by N atoms when there is only one Al or Cr atom replaced by TM atoms (shown in Figure 1c). However, it has previously been observed that replacing the Cr atom is energetically more favorable [16]. After that, supercells with higher TM contents when two adjacent Cr atoms are replaced by TM atoms can be established. This paper considers the situation where the second dopant atom is located at the nearest and second-nearest neighbor positions to the first dopant site. In both cases, the dopant atoms remain at the center of an octahedral composed of N atoms, indicating that there is only one configuration for the nearest and next-nearest neighbors, as shown in Figure 1c and 1d, respectively.

In this study, we initially calculated the enthalpy of formation of two models with Ti atoms as dopants. The enthalpy of formation for the configurations replacing the nearest and next-nearest neighbors was found to be −86.69 and −86.56 kJ/mol-atom, respectively. The formation enthalpies of the two models are very close, with the nearest neighbor configuration being relatively lower. Therefore, we proceed with the calculation for the case where two dopant atoms replace two Cr atoms in a nearest-neighbor arrangement.

First-principles calculations in the present work were performed based on the pseudo-potential plane wave within the density functional theory (DFT) by means of the Vienna Ab initio Simulation Package (VASP) [42,43]. After the convergence test, the ion–electron interaction and the exchange correlation effects were evaluated by the projector augmented wave method (PAW) [44] with an energy cutoff of 500 eV and the generalized gradient approximation (GGA) parameterized by Perdew–Burke–Ernzerhof (PBE) [45]. The k-points mesh of the Brillouin zone sampling was fixed at 7 × 7 × 7 using the Monk–horst–Pack scheme [46]. The total energy was converged to 0.01 meV/atom and 0.001 meV/atom by the tetrahedron method with Blöchl corrections [47] and the structure relaxation was performed by the conjugate gradient algorithm until the residual forces acting on ions are smaller than 0.02 eV/Å and 0.01 eV/Å for structure optimization and elastic properties calculation, respectively. In addition, the selected valence electrons were 3p^6^3d^5^4s^1^ for Cr, 3s^2^3p^1^ for Al, 2s^2^2p^3^ for N, 3p^6^3d^2^4s^2^ for Ti, 3s^2^3p^1^ for V, 4s^2^4p^6^4d^1^5s^2^ for Y, 4s^2^4p^6^4d^2^5s^2^ for Zr, 5p^6^5d^2^6s^2^ for Hf, and 5p^6^5d^3^6s^2^ for Ta. All calculations utilized the spin polarization approach.

The 4-parameter Birch–Murnaghan equation of state (EOS) was applied to fit the energy vs. volume (*E*-*V*) of first-principles calculation data points to estimate the ground state properties [48,49]:

(1) $$E\left(V\right)=a+bV^{-\frac{2}{3}}+cV^{-\frac{4}{3}}+{dV}^{-\frac{6}{3}}$$

where *a*, *b*, *c*, and *d* are fitting parameters. The EOS can be used to evaluate the structure properties, including the equilibrium total energy *E*_0_ and volume *V*_0_.

The enthalpy of formation signifies the alteration in energy that transpires under standard conditions as elemental components unite in their most stable configurations to produce a specific substance, either emitting or absorbing heat during the procedure. This serves as a crucial thermodynamic factor that delineates the stability and formation mechanism of materials, finding extensive application in both material science research and engineering pursuits [50,51]. Thermodynamic stability of Cr_0.5-x_Al_0.5_TM_x_N (TM = Ti, V, Y, Zr, Hf, and Ta) can be studied by the enthalpy of formation at 0 K, E*_f_* (kJ/mol), with the following equation:

(2) $$E_{f}=\frac{E_{bulk}({Cr}_{0.5\text{-}x}{Al}_{x}TM_{x}N)-32[(\left(\frac{1}{2}-x\right)E_{bulk}\left(Cr\right)-\frac{1}{2}E_{bulk}\left(Al\right)-xE_{bulk}\left(TM\right)-E_{bulk}\left(N\right)}{64}$$

where *E_bulk_* (Cr_0.5-x_Al_0.5_TM_x_) is the total energy of Cr_0.5-x_Al_0.5_TM_x_N, *E_bulk_* (TM) is the total energy per atom of Cr, Al, Ti, V, Y, Zr, Hf, Ta, and N of pure element in its ground state.

### 2.2. Elastic Properties

In the present work, the elastic stiffness constants were calculated by the strain–stress method demonstrated by Shang [40,52,53]. In this methodology, a set of strains *ε* with the normal strains (*ε*_1_, *ε*_2_, *ε*_3_) and shear strains (*ε*_4_, *ε*_5_, *ε*_6_) are imposed on a crystal by specifying the lattice vectors ***R*** in Cartesian coordinates, as follows:

(3) $$R=\left[\begin{array}{ccc} a_{1} & a_{2} & a_{3}\\ b_{1} & b_{2} & b_{3}\\ c_{1} & c_{2} & c_{3} \end{array}\right]$$

where $\bar{a}=(a_{1},a_{2},a_{3})$, $\bar{b}=\left(b_{1},b_{2},b_{3}\right),$ and $\bar{c}=(c_{1},c_{2},c_{3})$ are the lattice vectors. After deformation ($\bar{R}$), the crystal vectors can be described as:

(4) $$\bar{R}=R\left[\begin{array}{ccc} 1+\varepsilon_{1} & \varepsilon_{6}/2 & \varepsilon_{5}/2\\ \varepsilon_{6}/2 & 1+\varepsilon_{2} & \varepsilon_{4}/2\\ \varepsilon_{5}/2 & \varepsilon_{4}/2 & 1+\varepsilon_{3} \end{array}\right]$$

Then, the linearly independent sets of strains are applied as follows:

(5) $$\left[\begin{array}{cccccc} s & 0 & 0 & 0 & 0 & 0\\ 0 & s & 0 & 0 & 0 & 0\\ 0 & 0 & s & 0 & 0 & 0\\ 0 & 0 & 0 & s & 0 & 0\\ 0 & 0 & 0 & 0 & s & 0\\ 0 & 0 & 0 & 0 & 0 & s \end{array}\right]$$

The strain *s* was set to ±0.01 and each row is one set of strains. Through first-principles calculations, the corresponding set of stresses ($\sigma =\sigma_{1},\sigma_{2},\sigma_{3},\sigma_{4},\sigma_{5},\sigma_{6})$) of the deformed crystal could be obtained. Based on general Hook’s law, the elastic stiffness constants (*C*_ij_) are acquired:

(6) $$\left[\begin{array}{cccccc} C_{11} & C_{12} & C_{13} & C_{14} & C_{15} & C_{16}\\ C_{21} & C_{22} & C_{23} & C_{24} & C_{25} & C_{26}\\ C_{31} & C_{32} & C_{33} & C_{34} & C_{35} & C_{36}\\ C_{41} & C_{42} & C_{43} & C_{44} & C_{45} & C_{46}\\ C_{51} & C_{52} & C_{53} & C_{54} & C_{55} & C_{56}\\ C_{61} & C_{62} & C_{63} & C_{64} & C_{65} & C_{66} \end{array}\right]=\varepsilon^{-1}\sigma$$

The NaCl-type FCC unit cell consisted of 3 independent single crystal elastic stiffness constants (*C*_11_, *C*_12_, and *C*_44_). The bulk modulus (*B*), shear modulus (*G*), Cauchy pressure (*P*_C_), and Zener’s anisotropy (*A*) can be computed by the Voigt–Reuss–Hill (VRH) approach based on these elastic stiffness constants [54]:

(7) $$B=B_{V}=B_{R}=\frac{1}{3}(C_{11}+2C_{12})$$

(8) $$G_{V}=\frac{\left(C_{11}+C_{12}+3C_{44}\right)}{5}$$

(9) $$G_{R}=\frac{5(C_{11}-C_{12})C_{44}}{4C_{44}+3(C_{11}-C_{12})}$$

(10) $$G=\frac{G_{V}+G_{R}}{2}$$

(11) $$P_{C}=C_{12}-C_{44}$$

(12) $$A=\frac{2C_{44}}{C_{11}-C_{12}}$$

The theoretical hardness H_V_ [55], Young’s modulus (*E*), and Poisson’s ratio (*v*) can be further computed by the bulk modulus (*B*) and shear modulus (*G*):

(13) $$H_{V}=(2\times {\left(\frac{G^{3}}{B^{2}}\right)}^{0.585})-3$$

(14) $$E=\frac{9BG}{3B+G}$$

(15) $$v=\frac{3B-2G}{2(3B+G)}$$

The mechanical stability of a cubic crystal can be confirmed by the Born stability criteria [56]:

(16) $$C_{44}>0;C_{11}-\left|C_{12}\right|>0;C_{11}+2C_{12}>0$$

## 3. Results and Discussion

### 3.1. Ground State Properties

**Figure 2:**
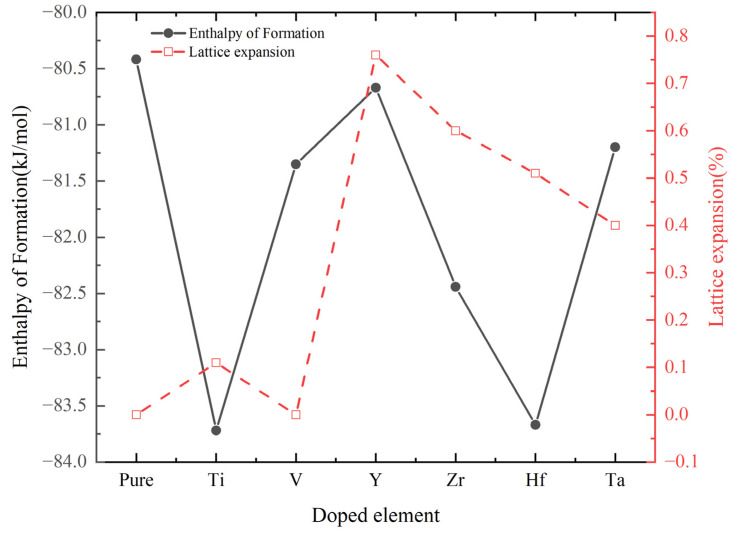
Volume and enthalpy of formations of Cr_0.47_Al_0.5_TM_0.03_N (TM = Ti, V, Y, Zr, Hf, and Ta).

Fitting the energy–volume data points with Equation (1), the first-principles calculated structure properties and enthalpy of formation of doping one TM (TM = Ti, V, Y, Zr, Hf and Ta) atom in the unit cell are shown in Table 1. For a clear comparison, Figure 2 summarizes the calculated values of structural properties and enthalpy of formation. It can be seen that the atomic radius is the main factor affecting the lattice parameters, the atomic radius of Y is the largest among the dopant elements, the volume of Cr_0.47_Al_0.5_Y_0.03_N is the largest. The volume and lattice constant of the unit cell expand due to the dopant elements, except for V. The lattice parameter 4.115 Å of Cr_0.36_Al_0.62_Ta_0.02_N calculated by Hollerweger [24] is close to the calculated value in this paper. The chemical stability of solid solution Cr_0.5-x_Al_0.5_TM_x_N (TM = Ti, V, Y, Zr, Hf, and Ta) can be studied by the enthalpy of formation with Equation (2). Among the doped compounds, the most energetically favorable doping is observed when Ti is introduced into Cr_0.5_Al_0.5_N.

**Figure 3:**
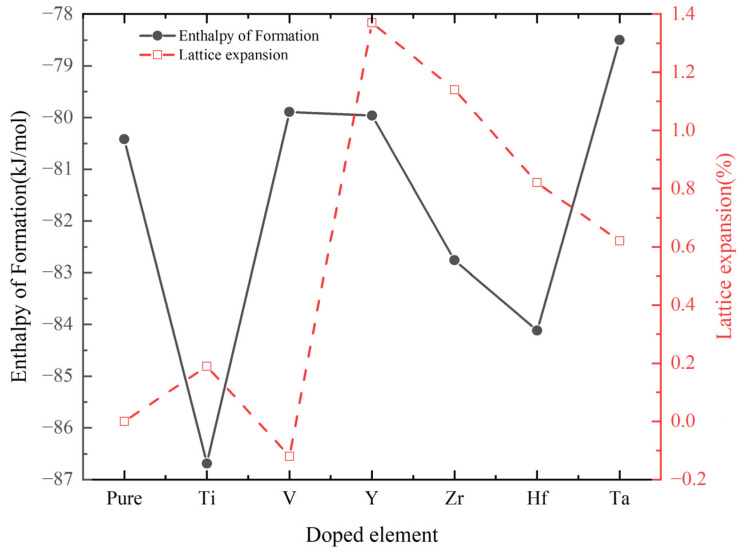
Volume and enthalpy of formations of Cr_0.44_Al_0.5_TM_0.06_N (TM = Ti, V, Y, Zr, Hf, and Ta).

Further increasing the solid solution amount of TM, the corresponding first-principles calculated structure properties and enthalpy of formation are listed in Table 2 and Figure 3. From the above calculation results, compared with Cr_0.5_Al_0.5_N, except that V addition shrinks the lattice, the lattice will expand when the solid solution amount of TM further increases. The primary reason for this phenomenon is the difference in atomic radii. Among the six elements Cr, V, Ti, Y, Hf, and Zr, V has the smallest atomic radius, measuring 1.34 Å, which is smaller than Cr’s 1.36 Å. On the other hand, the atomic radii of Ti, Y, Hf, and Zr are all larger than Cr’s atomic radius. Notably, Y has the largest atomic radius at 1.80 Å, making the lattice expansion percentage of Cr_0.44_Al_0.5_Y_0.06_N the highest, reaching 1.37. The lattice constant of the experimentally prepared Cr_0.44_Al_0.5_Ta_0.06_N is 4.117 Å, which is in good agreement with the calculated value 4.121 Å in this paper. Moreover, Cr_0.44_Al_0.5_Ti_0.06_N is energetically more favorable among doped compounds.

### 3.2. Elastic Properties

**Figure 4:**
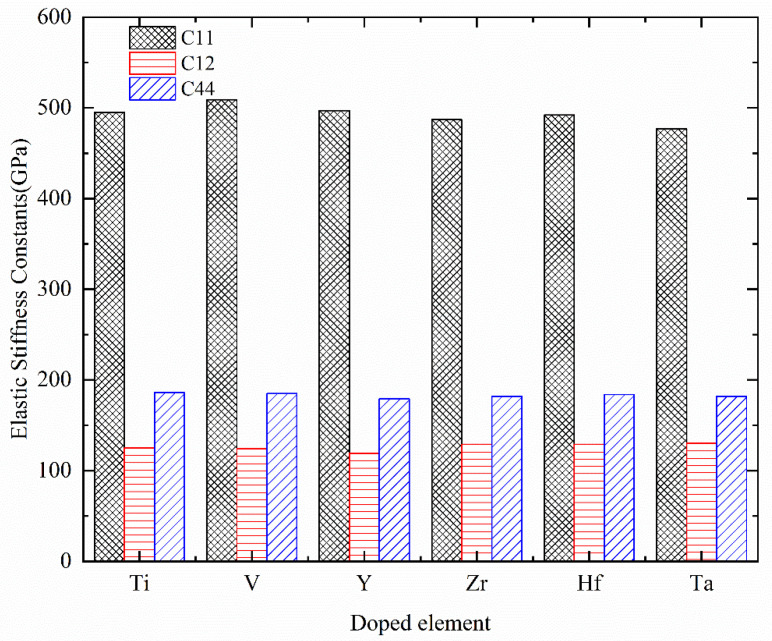
The histogram of variations in elastic stiffness constants of Cr_0.47_Al_0.5_TM_0.03_N with TM = Ti, V, Y, Zr, Hf, and Ta.

Table 3 summarizes the first-principles calculated elastic stiffness constants *C*_ij_ of Cr_0.47_Al_0.5_TM_0.03_N, where TM = Ti, V, Y, Zr, Hf, and Ta. Additionally, a histogram illustrating the variation of elastic stiffness constants is presented in Figure 4. It can be observed that the results of Cr_0.5_Al_0.5_N are in good agreement with the theoretically predicted values [57], and only some discrepancies exist. Considering the deviation in the research method, the discrepancy is acceptable.

Based on the mechanical stability criteria in Equation (16) at a given pressure, the computed results indicate that the TM solid solution does not destroy the mechanical stability of Cr_0.5_Al_0.5_N. The elastic stiffness constant *C*_11_ describes the resistance to linear compression along crystallographic *a*, *b*, *c* axes. *C*_12_ explains the resistance to the strain along crystallographic b axes when a stress is applied in the crystallographic direction. *C*_44_ demonstrates the resistance to the shear deformation in (100) plane. Elastic stiffness constant *C*_11_ of Cr_0.5_Al_0.5_N and Cr_0.47_Al_0.5_TM_0.03_N (TM = Ti, V, Y, Zr, Hf, and Ta) is significantly stiffer than the other two elastic stiffness constants. From results in our work, TM (TM = Ti, V, Y, Zr, Hf, and Ta) doping could decrease *C*_11_ value for Cr_0.5_Al_0.5_N. All Cr_0.47_Al_0.5_TM_0.03_N compounds possess higher *C*_12_ values than Cr_0.5_Al_0.5_N except for Cr_0.47_Al_0.5_Y_0.03_N, which possesses the lowest *C*_12_ value among all compounds. Among doped compounds, Cr_0.47_Al_0.5_V_0.03_N possesses the largest *C*_11_ value, that means it is hardest to compress along crystallographic axes. Cr_0.47_Al_0.5_Ta_0.03_N possesses the largest *C*_12_ value but lowest *C*_11_ value.

**Figure 5:**
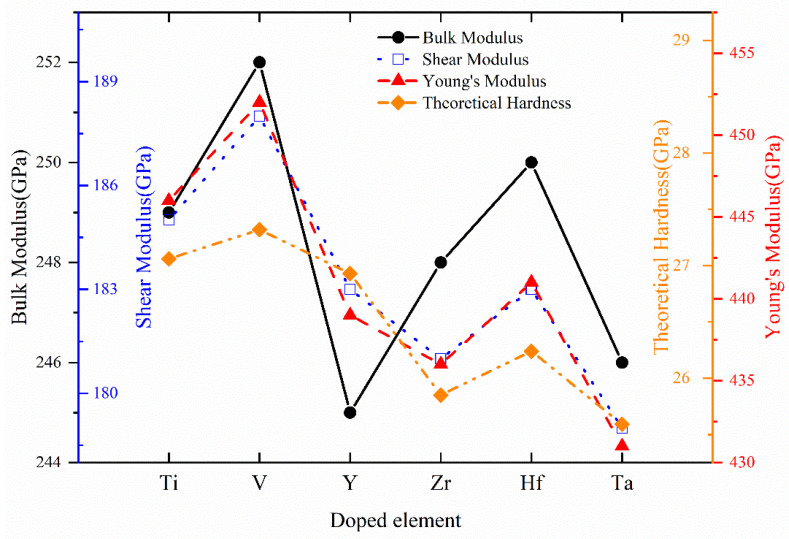
The variation trend of bulk modulus, shear modulus, Young’s modulus, and theoretical hardness of Cr_0.47_Al_0.5_TM_0.03_N with TM = Ti, V, Y, Zr, Hf, and Ta.

According to elastic stiffness constants, the aggregate polycrystalline mechanical properties such as bulk modulus *B*, shear modulus *G*, Young’s modulus *E*, Poisson’s ratio *v*, Pugh’s ratio *B*/*G*, Zener’s anisotropy A, and theoretical hardness *H*_V_ of Cr_0.5_Al_0.5_N and Cr_0.47_Al_0.5_TM_0.03_N (TM = Ti, V, Y, Zr, Hf, and Ta) are calculated and listed in Table 4. For convenience of comparison, the variation trends of bulk modulus, shear modulus, Young’s modulus, and theoretical hardness are shown in Figure 5. The bulk modulus *B* reflects the resistance to compression and the strength of the chemical bond, which is defined as the ratio of the change in pressure to the fractional volume compression. According to Table 4 and Figure 5, Cr_0.5_Al_0.5_N and all Cr_0.47_Al_0.5_TM_0.03_N (TM = Ti, V, Y, Zr, Hf, and Ta) compounds possess a comparatively high bulk modulus, which reflects great resistance to volume deformation and strong chemical bond strength in the crystal. All Cr_0.47_Al_0.5_TM_0.03_N (TM = Ti, V, Y, Zr, Hf, and Ta) compounds possess lower bulk modulus than Cr_0.5_Al_0.5_N. Cr_0.47_Al_0.5_V_0.03_N possesses the highest bulk modulus among doped compounds, which implies that it has a greater rigidity. Generally, the bulk modulus is also used to measure the ability of materials to resist external forces [58]. Therefore, we can know the order of the ability to resist external forces according to the order of bulk modulus values from small to large as follows: Cr_0.47_Al_0.5_V_0.03_N > Cr_0.47_Al_0.5_Ti_0.03_N > Cr_0.47_Al_0.5_Hf_0.03_N > Cr_0.47_Al_0.5_Zr_0.03_N > Cr_0.47_Al_0.5_Ta_0.03_N > Cr_0.47_Al_0.5_Y_0.03_N.

The shear modulus describes the resistance to the plastic deformation of a material. From Table 4, the shear modulus of Cr_0.5_Al_0.5_N is higher than all doped compounds, which means it could possess better ability against the shear force. Among the doped compounds, Cr_0.47_Al_0.5_V_0.03_N and Cr_0.47_Al_0.5_Ta_0.03_N possess the highest and lowest shear modulus, respectively. The Young’s modulus *E* measures the tensile or compressive stiffness of a solid material when the force is applied lengthwise [59], and it can reflect the relationship of plastic; the smaller the Young’s modulus, the more prone to plastic deformation. It can be observed that TM (TM = Ti, V, Y, Zr, Hf, and Ta) doping could decrease the Young’s modulus for Cr_0.5_Al_0.5_N, especially Ta. Cr_0.47_Al_0.5_V_0.03_N possesses the highest Young’s modulus among doped compounds, indicating greater stiffness and good ability to resist longitudinal tensions. The order of Young’s modulus values from small to large is Cr_0.47_Al_0.5_Ta_0.03_N < Cr_0.47_Al_0.5_Zr_0.03_N < Cr_0.47_Al_0.5_Y_0.03_N < Cr_0.47_Al_0.5_Hf_0.03_N < Cr_0.47_Al_0.5_Ti_0.03_N < Cr_0.47_Al_0.5_V_0.03_N. Shear modulus *G* and Young’s modulus *E* can assess the hardness and stiffness of materials to a certain extent, and are positively correlated with each other [58]. Therefore, the order of *G* and *E* values from large to small is: Cr_0.47_Al_0.5_V_0.03_N > Cr_0.47_Al_0.5_Ti_0.03_N > Cr_0.47_Al_0.5_Hf_0.03_N > Cr_0.47_Al_0.5_Y_0.03_N > Cr_0.47_Al_0.5_Zr_0.03_N > Cr_0.47_Al_0.5_Ta_0.03_N, Cr_0.47_Al_0.5_V_0.03_N, which could have the greatest stiffness and hardness among doped compounds. Other studies [16,24] also found that doping Y, Ta, and V could decrease bulk modulus and Young’s modulus, and doping V could promote the formation of the hexagonal phase of the metastable cubic lattice, resulting in a decrease in hardness, which is consistent with our findings. In summary, within the concentration range studied in this paper, the calculated results can evaluate the influence of transition metal elements TM (TM = Ti, V, Y, Zr, Hf, and Ta) on the mechanical properties of c-Cr_0.5_Al_0.5_N.

Cauchy pressure *P*_c_ (*C*_12_–*C*_44_) is used to evaluate the bond type, in which a more negative value indicates a stronger and more directional covalent bond. Therefore, among the doped compounds, Cr_0.47_Al_0.5_TM_0.03_N with Ti, and V could exhibit more significant directional covalent bonding with a more negative value of Cauchy pressure, which results in an increased resistance against shearing. Poisson’s ratio (*v*) is a measure of the deformation of a material in directions perpendicular to the specific direction of stress, which is generally used to represent the shear resistance of the material. Poisson’s ratio is also the characteristic of atomic forces inside the material, if the value of Poisson’s ratio is within 0.25 and 0.5, the material can be considered as a central force solid; otherwise, it is non-central force solid [59,60]. If *v* = 0.5, no volume change occurs during elastic deformation [61]. In the calculation, the Poisson’s ratio values for Cr_0.5_Al_0.5_N and Cr_0.47_Al_0.5_TM_0.03_N (TM = Ti, V, Y, Zr, Hf, and Ta) are all around 0.20, as listed in Table 4, which means that all of them are a non-central force solid and the low *v* value shows that the considerable volume change occurs during deformation. Additionally, Cauchy pressure (*P*_c_), Poisson’s ratio (*v*), and Pugh’s index of ductility (*B*/*G*) provide information about the failure model of solid. The material is considered as brittle when the value of Cauchy pressure is negative, Poisson’s ratio is smaller than 0.26, and *B*/*G* < 1.75 or *G*/*B* > 0.571. According to this criterion, Cr_0.5_Al_0.5_N and all doped compounds Cr_0.47_Al_0.5_TM_0.03_N (TM = Ti, V, Y, Zr, Hf, and Ta) can be regarded as brittle material.

When the elastic anisotropy of a material is strong, the greater the difference in its ability to resist deformation in different directions and more prone to deformation fracture. For the cubic system, Zener’s anisotropy A can reflect the elastic anisotropy of materials. When the material is isotropic, *A* = 1; otherwise, the materials are anisotropic. The more Zener’s anisotropy deviates from 1, the more elastic anisotropy the crystalline structure has. The calculation result of Zener’s elastic anisotropy indicates a comparatively weak elastic anisotropy in both Cr_0.5_Al_0.5_N and Cr_0.47_Al_0.5_TM_0.03_N (TM = Ti, V, Y, Zr, Hf, and Ta). Moreover, Zener’s anisotropy accounts for the degree of dielectric breakdown and the resistance of microcracks [40,62]. It is found that Cr_0.47_Al_0.5_Ta_0.03_N exhibits the highest degree of dielectric breakdown and the lowest resistance of microcracks due to the largest Zener’s anisotropy. The calculated theoretical hardness HV of all compounds is also listed in Table 3. It could be found that TM (TM = Ti, V, Y, Zr, Hf, and Ta) doping could decrease the theoretical hardness for Cr_0.5_Al_0.5_N, especially Ta. Among all doped compounds, Cr_0.47_Al_0.5_V_0.03_N possesses the highest values for bulk modulus, shear modulus, Young’s modulus, and theoretical hardness.

**Figure 6:**
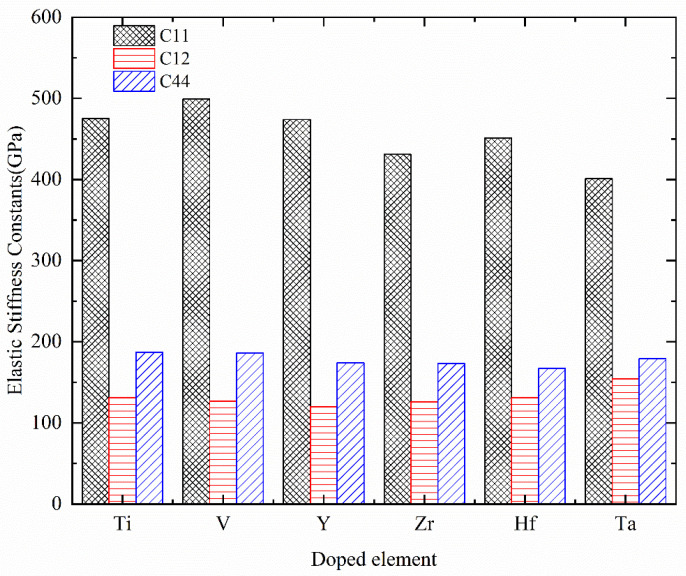
The histogram of variations in elastic stiffness constants of Cr_0.44_Al_0.5_TM_0.06_N with TM = Ti, V, Y, Zr, Hf, and Ta.

When Cr atoms are stepwise substituted by TM (TM = Ti, V, Y, Zr, Hf, and Ta) atoms, the calculated elastic stiffness constants *C*_ij_ of Cr_0.44_Al_0.5_TM_0.06_N are presented in Table 5 and plotted in Figure 6. The evaluation of these elastic stiffness constants is crucial for understanding the mechanical stability of the doped compounds. According to the results in Table 5, all doped compounds are mechanically stable. From a comparative analysis with Cr_0.47_Al_0.5_TM_0.03_N, the elastic stiffness constant *C*_11_ of Cr_0.44_Al_0.5_TM_0.06_N significantly decreases, which indicates that the axial compression resistance of the doped compounds decreases with the increase in TM amount. Furthermore, the variations in *C*_12_ differ among the different Cr_0.44_Al_0.5_TM_0.06_N compounds, with an overall increase observed, except for the case of Zr doping. This indicates a complex interplay between the substitution of Cr by different TM elements and their impact on the material’s resistance to deformation under specific loading conditions. Additionally, only the elastic stiffness constant *C*_44_ of Cr_0.47_Al_0.5_TM_0.03_N exhibits an increase with TM = Ti and V, highlighting the nuanced effects of TM doping on the mechanical properties. These insights into the changes in elastic stiffness constants provide valuable information about the mechanical response of Cr_0.44_Al_0.5_TM_0.06_N to TM doping. Understanding these variations is crucial for tailoring the material’s properties to meet specific performance requirements in diverse applications.

**Figure 7:**
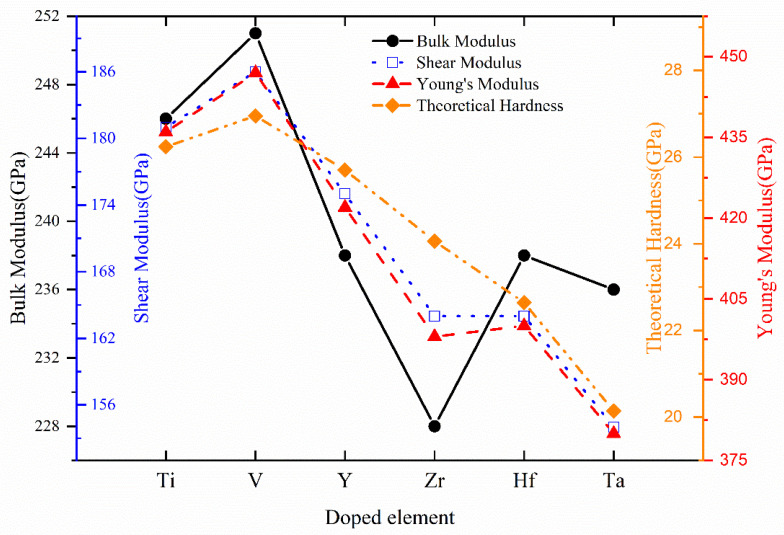
The variation trend of bulk modulus, shear modulus, Young’s modulus, and theoretical hardness of Cr_0.44_Al_0.5_TM_0.06_N with TM = Ti, V, Y, Zr, Hf, and Ta.

Appling VRH approximation from elastic stiffness constants, bulk modulus *B*, shear modulus *G*, Young’s modulus *E*, Poisson’s ratio *v*, Pugh’s index of ductility *B*/*G*, Zener’s anisotropy A, and theoretical hardness *H_V_* of Cr_0.5_Al_0.5_N and Cr_0.44_Al_0.5_TM_0.06_N (TM = Ti, V, Y, Zr, Hf, and Ta) are calculated and presented in Table 6. To provide a visual representation of the variation trends, Figure 7 illustrates the variation trend of bulk modulus, shear modulus, Young’s modulus, and theoretical hardness across the different compositions. According to Table 3 and Table 6, with the increase in TM addition, the mechanical properties of Cr_0.47_Al_0.5_TM_0.03_N such as bulk modulus, shear modulus, Young’s modulus, and theoretical hardness exhibit a decreasing trend, while an increase in Cauchy pressure and Pugh’s index of ductility indicates an increased toughness and a tendency toward the mobile character of the bonds, which results in a decreased resistance against shearing. It is also found that there is almost no change in the increase in Poisson’s ratio and Zener’s anisotropy with the increasing mole fraction of TM. In summary, the presented data shed light on the nuanced variations in mechanical properties induced by the introduction of different transition metals in Cr_0.44_Al_0.5_TM_0.06_N and Cr_0.47_Al_0.5_TM_0.03_N. These findings contribute to a deeper understanding of the structure-property relationships, offering valuable insights for optimizing the material’s performance in diverse engineering and technological applications.

### 3.3. Electronic Properties

**Figure 8:**
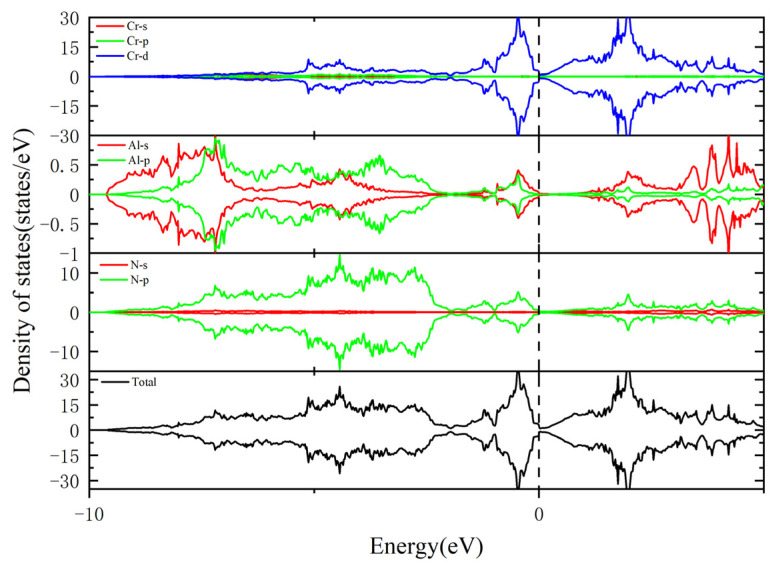
Total and partial density of states of Cr_0.5_Al_0.5_N. The vertical dashed line represents the Fermi level.

**Figure 9:**
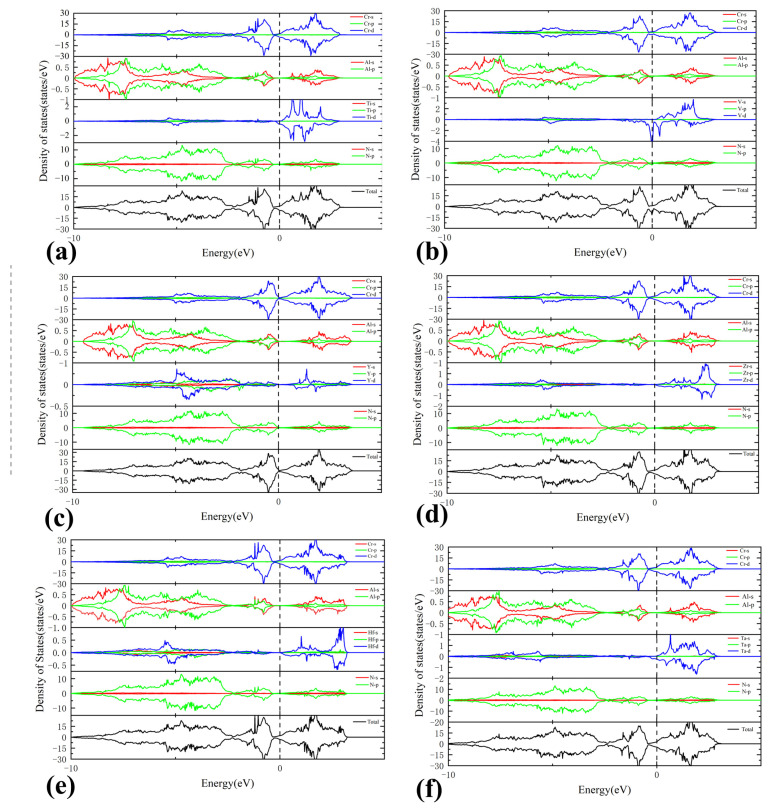
Total and partial density of states. (**a**) Cr_0.47_Al_0.5_Ti_0.03_N, (**b**) Cr_0.47_Al_0.5_V_0.03_N, (**c**) Cr_0.47_Al_0.5_Y_0.03_N, (**d**) Cr_0.47_Al_0.5_Zr_0.03_N, (**e**) Cr_0.47_Al_0.5_Hf_0.03_N, and (**f**) Cr_0.47_Al_0.5_Ta_0.03_N. The vertical dashed line represents the Fermi level.

The total and partial density of states (TDOS and PDOS, respectively) of Cr_0.5_Al_0.5_N and Cr_0.47_Al_0.5_TM_0.03_N (TM = Ti, V, Y, Zr, Hf, and Ta) were calculated and presented in Figure 8 and Figure 9. The dashed lines denote the Fermi level which is 0 eV. It is found that due to the introduction of impurity atoms, the density of states moves to the lower energy level, indicating the existence of electron transfer between atoms. All compounds have some finite DOS around the Fermi level, which is mainly contributed by the Cr-d and some of the TM-d states, indicating the metallic characteristic. Additionally, the PDOS analysis reveals intricate details about the contribution of specific orbitals to the electronic structure.

Moreover, except for Cr atoms, the contribution of Ti-3d, V-3d, and Ta-3d at the Fermi level is significantly higher than that of other TM atoms, leading to the enhanced metallic character of Cr_0.47_Al_0.5_Ti_0.03_N, Cr_0.47_Al_0.5_V_0.03_N, and Cr_0.47_Al_0.5_Ta_0.03_N. This heightened contribution from specific orbitals not only influences the metallic behavior, but also contributes to the overall electronic properties of the doped compounds. The peak near the Fermi energy level of total state density maps of Cr_0.47_Al_0.5_TM_0.03_N is similar to Cr_0.5_Al_0.5_N, which means that the low concentration of transition metal doping dose not significantly change the metal properties and structural stability of Cr_0.5_Al_0.5_N. Therefore, the model structures used in this paper are stable, providing a reliable foundation for further investigations into the material’s electronic and structural characteristics. This paper employs PBE for calculating electronic properties, but it is important to note that PBE may underestimate bandgaps. One corrective approach is the utilization of hybrid functionals and self-interaction corrected methods, which can provide more accurate band structures [63,64]. This consideration was not taken into account in the current study and will be addressed in future research for improvement.

## 4. Conclusions

Based on the first-principles calculations, 2 × 2 × 2 supercells of Cr_0.47_Al_0.5_TM_0.03_N and Cr_0.44_Al_0.5_TM_0.06_N (TM = Ti, V, Y, Zr, Hf, and Ta) were meticulously constructed to study their intricate structural, mechanical, and electronic properties. Analysis of the enthalpy of formation indicates that all doped compounds are thermodynamically stable within the studied conditions. The meticulous examination of these stability aspects establishes a robust foundation for the subsequent investigations into the unique characteristics of each doped material. The lattice and volume of Cr_0.5-x_Al_0.5_TM_x_N expand with the addition of Ti, Y, Zr, Hf, and Ta, while V exhibits a contrary effect.

Furthermore, a comprehensive evaluation of the mechanical properties was performed, including elastic stiffness constants, Cauchy pressure, bulk modulus, shear modulus, Young’s modulus, Poisson’s ratio, Pugh’s index of ductility, Zener’s anisotropy, and theoretical hardness, and the results were discussed. The mechanical properties of Cr_0.5_Al_0.5_N were not improved compared with Cr_0.47_Al_0.5_TM_0.03_N and Cr_0.44_Al_0.5_TM_0.06_N. Among the doping compounds at the same doping concentration, Cr_0.5-x_Al_0.5_V_x_N exhibited the highest ability to resist external force, as well as the highest stiffness and hardness. Cr_0.5-x_Al_0.5_Ta_x_N demonstrated the highest plasticity, exhibiting the highest degree of dielectric breakdown and the lowest resistance of microcracks due to the largest Zener’s anisotropy.

The mechanical properties of Cr_0.5-x_Al_0.5_TM_x_N were found to decrease, while the toughness increased as Cr atoms were stepwise substituted by TM atoms. The finite density of states (DOS) at the Fermi level indicates the metallic behavior of Cr_0.5-x_Al_0.5_TM_x_N. It was observed that the DOS of Cr_0.5-x_Al_0.5_TM_x_N is similar to Cr_0.5_Al_0.5_N, suggesting that the addition of elements with low doping concentration has little effect on the structural stability of Cr_0.5-x_Al_0.5_TM_x_N. The contribution of Ti-3d, V-3d, and Ta-3d orbitals to the density of states (DOS) at the Fermi level was observed to be higher than that of other dopant elements. This enhancement in orbital contribution is associated with the enhanced metallic character of Cr_0.47_Al_0.5_Ti_0.03_N, Cr_0.47_Al_0.5_V_0.03_N, and Cr_0.47_Al_0.5_Ta_0.03_N.

In summary, the extended investigations into the structural, mechanical, and electronic properties of Cr_0.5-x_Al_0.5_TM_x_N, guided by first-principles calculations, provide a comprehensive understanding of the intricate interplay between dopant elements and material characteristics. These findings not only contribute to the fundamental knowledge of alloy systems, but also pave the way for the design and optimization of materials with tailored properties for diverse technological applications.

## Figures and Tables

**Table 1:** Calculated lattice parameter and stability information of Cr_0.47_Al_0.5_TM_0.03_N with TM = Ti, V, Y, Zr, Hf, and Ta.

System	Volume (Å^3^)	Lattice Constant (Å)	Lattice Expansion (%)	Enthalpy of Formation (kJ/mol)
Cr_0.5_Al_0.5_N	562.19	8.253	0	−80.42
Cr_0.47_Al_0.5_Ti_0.03_N	564.06	8.262	0.11	−83.72
Cr_0.47_Al_0.5_V_0.03_N	562.07	8.253	0	−81.35
Cr_0.47_Al_0.5_Y_0.03_N	575.06	8.316	0.76	−80.67
Cr_0.47_Al_0.5_Zr_0.03_N	572.14	8.302	0.60	−82.44
Cr_0.47_Al_0.5_Hf_0.03_N	570.72	8.293	0.51	−83.67
Cr_0.47_Al_0.5_Ta_0.03_N	568.89	8.286	0.40	−81.20

**Table 2:** Calculated lattice parameter and stability information of Cr_0.44_Al_0.5_TM_0.06_N with TM = Ti, V, Y, Zr, Hf, and Ta.

Compound	Volume (Å^3^)	Lattice Constant (Å)	Lattice Expansion (%)	Enthalpy of Formation (kJ/mol)
Cr_0.5_Al_0.5_N	562.19	8.253	0	−80.42
Cr_0.44_Al_0.5_Ti_0.06_N	565.49	8.269	0.19	−86.69
Cr_0.44_Al_0.5_V_0.06_N	560.40	8.243	−0.12	−79.89
Cr_0.44_Al_0.5_Y_0.06_N	585.45	8.366	1.37	−79.96
Cr_0.44_Al_0.5_Zr_0.06_N	581.51	8.347	1.14	−82.76
Cr_0.44_Al_0.5_Hf_0.06_N	576.07	8.321	0.82	−84.12
Cr_0.44_Al_0.5_Ta_0.06_N	572.57	8.304	0.62	−78.50

**Table 3:** Calculated elastic constants *C*_ij_ (GPa), Cauchy pressure *Pc* (*C*_12_–*C*_44_) (GPa), and mechanical stability of Cr_0.5_Al_0.5_N and Cr_0.47_Al_0.5_TM_0.03_N with TM = Ti, V, Y, Zr, Hf, and Ta.

System	*C*_11_	*C*_12_	*C*_44_	*C*_12_–*C*_44_	Mechanical Stability
Cr_0.5_Al_0.5_N	519	120	184	−64	Stable
Cr_0.5_Al_0.5_N	502	123	175	−52	Stable
Cr_0.5_Al_0.5_N	522	121	184	−63	Stable
Cr_0.47_Al_0.5_Ti_0.03_N	495	125	186	−61	Stable
Cr_0.47_Al_0.5_V_0.03_N	509	124	185	−61	Stable
Cr_0.47_Al_0.5_Y_0.03_N	497	119	179	−60	Stable
Cr_0.47_Al_0.5_Zr_0.03_N	487	129	182	−53	Stable
Cr_0.47_Al_0.5_Hf_0.03_N	492	129	184	−55	Stable
Cr_0.47_Al_0.5_Ta_0.03_N	477	130	182	−52	Stable

**Table 4:** Calculated mechanical properties including bulk modulus *B* (GPa), shear modulus *G* (GPa), Young’s modulus *E* (GPa), Poisson’s ratio v, Pugh’s index of ductility *B*/*G*, Zener’s anisotropy *A*, and theoretical hardness *H*_V_ (GPa) of Cr_0.47_Al_0.5_TM_0.03_N (TM = Ti, V, Y, Zr, Hf, and Ta).

System	*B*_V_	*B*_R_	*B*	*G*_V_	*G*_R_	*G*	*E*	*v*	*B*/*G*	*A*	*H_V_*
Cr_0.5_Al_0.5_N	253	253	253	190	190	190	456	0.20	1.33	0.92	27.82
Cr_0.47_Al_0.5_Ti_0.03_N	249	249	249	185	185	185	446	0.20	1.34	1.01	27.06
Cr_0.47_Al_0.5_V_0.03_N	252	252	252	188	188	188	452	0.20	1.34	0.96	27.32
Cr_0.47_Al_0.5_Y_0.03_N	245	245	245	183	183	183	439	0.20	1.34	0.95	26.93
Cr_0.47_Al_0.5_Zr_0.03_N	248	248	248	181	181	181	436	0.21	1.37	1.02	25.85
Cr_0.47_Al_0.5_Hf_0.03_N	250	250	250	183	183	183	441	0.20	1.37	1.01	26.24
Cr_0.47_Al_0.5_Ta_0.03_N	246	246	246	179	179	179	431	0.21	1.38	1.05	25.59

**Table 5:** Calculated elastic constants *C*_ij_ (GPa), Cauchy pressure *P*_c_ (*C*_12_–*C*_44_) (GPa), and mechanical stability of Cr_0.44_Al_0.5_TM_0.06_N with TM = Ti, V, Y, Zr, Hf, and Ta.

System	*C*_11_	*C*_12_	*C*_44_	*C*_12_–*C*_44_	Mechanical Stability
Cr_0.5_Al_0.5_N	519	120	184	−64	Stable
Cr_0.44_Al_0.5_Ti_0.06_N	475	131	187	−56	Stable
Cr_0.44_Al_0.5_V_0.06_N	499	127	186	−59	Stable
Cr_0.44_Al_0.5_Y_0.06_N	474	120	174	−54	Stable
Cr_0.44_Al_0.5_Zr_0.06_N	431	126	173	−47	Stable
Cr_0.44_Al_0.5_Hf_0.06_N	451	131	167	−36	Stable
Cr_0.44_Al_0.5_Ta_0.06_N	401	154	179	−25	Stable

**Table 6:** Calculated reference data of mechanical properties including bulk modulus *B* (GPa), shear modulus *G* (GPa), Young’s modulus *E* (GPa), Poisson’s ratio *v*, Pugh’s index of ductility *B*/*G*, Zener’s anisotropy *A*, and theoretical hardness *H_V_* (GPa) of Cr_0.44_Al_0.5_TM_0.06_N (TM = Ti, V, Y, Zr, Hf, and Ta).

System	*B*_V_	*B*_R_	*B*	*G*_V_	*G*_R_	*G*	*E*	*v*	*B*/*G*	*A*	*H_V_*
Cr_0.5_Al_0.5_N	253	253	253	190	190	190	456	0.20	1.33	0.92	27.82
Cr_0.44_Al_0.5_Ti_0.06_N	246	246	246	181	181	181	436	0.20	1.36	1.09	26.24
Cr_0.44_Al_0.5_V_0.06_N	251	251	251	186	186	186	447	0.20	1.35	1.00	26.95
Cr_0.44_Al_0.5_Y_0.06_N	238	238	238	175	175	175	422	0.20	1.36	0.98	25.70
Cr_0.44_Al_0.5_Zr_0.06_N	228	228	228	164	165	164	398	0.21	1.38	1.13	24.06
Cr_0.44_Al_0.5_Hf_0.06_N	238	238	238	164	164	164	400	0.22	1.45	1.04	22.64
Cr_0.44_Al_0.5_Ta_0.06_N	236	236	236	154	157	152	380	0.23	1.53	1.45	20.14

## Data Availability

Data are available within the article.
